# Factors influencing the utilization of dental services in East Java, Indonesia

**DOI:** 10.12688/f1000research.23698.2

**Published:** 2021-04-21

**Authors:** Ninuk Hariyani, Dini Setyowati, Multia Ranum Sari, Diah Ayu Maharani, Rahul Nair, Kaushik Sengupta

**Affiliations:** 1 Department of Dental Public Health, Faculty of Dental Medicine Universitas Airlangga, Surabaya, East Java, 60132, Indonesia; 2 Australian Research Centre for Population Oral Health, Adelaide Dental School, The University of Adelaide, Adelaide, South Australia, Australia; 3 Faculty of Dentistry, Bhakti Wiyata Institute of Health Sciences, Kediri, East Java, Indonesia; 4 Department of Preventive and Public Health Dentistry, Faculty of Dentistry, Universitas Indonesia, Jakarta, Indonesia; 5 Department of Dentistry - Quality and Safety of Oral Healthcare, Radboud University Medical Center, Radboud Institute for Health Sciences, Nijmegen, The Netherlands; 6 Department of Public Health, University of Copenhagen, Copenhagen, Denmark

**Keywords:** dental service utilization, edentulism, tooth brushing habits, Indonesian, population-based study

## Abstract

**Background** ***:***Despite high levels dental issues and insurance coverage in the East Java province Indonesia, the utilization of dental services is still low. This research aims to test whether certain individual-level sociodemographic, behavioural, and clinical characteristics influenced the current level of dental services utilization by East Java residents.

**Methods** ***:***A secondary analysis was undertaken using data on the East Java province from the Indonesian Basic Health Research 2013, which included 90,551 randomly selected respondents aged 5–100 years old. Socio-demographic characteristics (age, sex, education and residential location), dental behavior (tooth brushing habit), and clinical (dental) condition were self-reported through a questionnaire. Multivariable models were generated to estimate prevalence ratios (PR), and 95% confidence intervals (95% CI).

**Results** ***:***Prevalence of dental service utilization during the last 12 months in East Java province is only 9 %. Respondents 25–<50 years old showed the highest utilization of dental services. Being male, having lower education and living in a district (as opposed to municipalities) were indicators for having lower utilization of dental treatment (PR [95% CI] = 0.81 [0.79–0.84], PR [95% CI] = 0.89 [0.86–0.93] and PR [95% CI] = 0.91 [0.88–0.95], respectively). Respondents with poor tooth brushing habit showed lower utilization of dental services. Having teeth was associated with higher utilization of dental treatment (PR [95% CI] = 1.39 [1.16–1.66).

**Conclusions** ***:***Age, sex, education and residential location influence the utilization of dental services among Indonesia’s East Java residents. Poor tooth brushing habits and being edentulous are also indicators of lesser utilization. These results call for urgent public health interventions to increase equitable dental care services utilization.

## Introduction

Health is a fundamental right of every human being without discrimination related to race, religion, and socioeconomic status ( World Health Organization, 2015). Oral health is integral to the overall health of human beings ( Peres *et al.*, 2019; World Health Organization, 2020). However, in most countries, access to and utilization of oral health services are limited ( Glick *et al.*, 2012; Petersen, 2003; Watt *et al.*, 2019). Lack of access to such services can have a detrimental impact on people’s general health and quality of life ( Petersen, 2003). Tooth loss is mainly the result of accumulated dental diseases as a product of low utilization of dental services ( Petersen, 2003).

One of the commonly used indices to assess the utilization of dental services is the percentage of the population attending a dental visit in the previous year ( Bayat *et al.*, 2008). The utilization of dental services is varied across countries. In developing countries, the majority of people only visits the dentist for pain relief rather than preventive care ( Varenne *et al.*, 2006), while in developed countries about 40–80% of the adults visit a dentist in a given year ( Bayat *et al.*, 2008).

Previous research has indicated certain factors that influence dental service utilization ( Fonseca *et al.*, 2017; Machry *et al.*, 2013; Vieira *et al.*, 2019; Vujicic & Nasseh, 2014). For example, socio-demographic factors related to dental service use include age, sex, education, and residential location ( Fonseca *et al.*, 2017; Machry *et al.*, 2013; Vieira *et al.*, 2019). Moreover, poor health behaviors are usually clustered in the same person wherein a person with a bad tooth brushing habit also rarely accesses dental treatments ( Jordao *et al.*, 2018). Furthermore, dental clinical condition such as dental status (dentate vs edentulous) could influence the utilization of dental services as it differentiates the extent of dental treatment need. So far, utilization of dental services and factors related to it have been mainly reported in developed countries, while such reporting in developing countries has been limited.

Oral health is a much neglected field of research in developing countries, including in Indonesia. Indonesia is the fourth most populated country in the world after China, India, and the United States of America ( United Nation, 2019). Cases of dental caries in this country is high; for example, more than 88% of the population has been estimated to have experienced caries, with 45% having untreated caries ( The Ministry of Health, Republic of Indonesia, 2019). Currently, Indonesia is in the process of establishing universal health coverage through Jaminan Kesehatan Nasional (JKN), wherein basic dental health is included in the insurance coverage. This insurance covers seven services: 1. dental examination, medication and consultation, 2. premedication, 3. dental emergency, 4. extraction of deciduous or permanent tooth without difficulties, 5. medication after extraction, 6. glass ionomer and composite dental fillings resulting from disease (not cosmetic reasons), and 7. scaling (once a year) ( Social Insurance Administration Organization, 2020). This universal health coverage program was implemented as a capitation system whereby the dentists are paid a fixed amount for the number of people who were under their care ( Deloitte Indonesia, 2019). The participants in the insurance scheme include Contribution Assistance Recipients (“ *Penerima Bantuan Iuran*,” PBI) and non-PBI members ( Deloitte Indonesia, 2019). PBI include poorer citizen whose insurance is funded by the government through taxes. Non-PBI members include other citizens not categorized as poor, who need to subscribe to the insurance scheme by paying for it monthly (e.g., through deduction directly from their income) ( Deloitte Indonesia, 2019). A comprehensive assessment of the JKN program conducted by the Government of Indonesia in 2017 found that JKN has managed to bring 76% of Indonesia’s population under the program; this is considered an impressive coverage rate ( Social Insurance Administration Organization, 2019).

Despite the high level of dental problems and the high insurance coverage, the utilization of dental services among the Indonesian population is very low ( The Ministry of Health Republic of Indonesia, 2013b). Indonesian Basic Health Research (Riset Kesehatan Dasar/RISKESDAS) 2018 showed that only 8.1% of Indonesians used dental services ( The Ministry of Health Republic of Indonesia, 2013b). In Indonesia, East Java is the second most populated province with a slightly below average national dentist-population ratio ( The Ministry of Health Republic of Indonesia, 2013a). The insurance coverage in this province is 80%. However, the utilization of dental services in this province is similar to the national estimate which is 8.6% ( The Ministry of Health, Republic of Indonesia, 2013b). Understanding the factors influencing dental service utilization among East Java residents is needed as a fundamental step to develop policy to increase the utilization of the services.

This study aimed to explore the associations of socio-demographic characteristics, behavioral factors, and clinical condition with the utilization of dental services among East Java residents.

## Methods

### Study population and research design

This secondary data analysis used data from the 2013 Indonesian Basic Health Survey (Riskesdas 2013). Riskesdas 2013 was a cross sectional national survey. It was part of a serial Indonesian national basic health survey conducted every six years. As the latest Riskesdas data is not currently open to the public, the Riskesdas 2013 data was used in this analysis. Riskesdas 2013 used a three-stage, stratified cluster sampling design to select a representative sample of Indonesian residents. The sampling frame was households recorded in the 2010 bloc census database, revalidated by the 2013 enumerator team. Indonesia was stratified into metropolitan and non-metropolitan areas by provincial status, with clusters based on district or municipality, which were selected with probability proportional to size. All persons in the household were included in the census. Final respondents were 294,959 households with the mean number of residents equal to 3.8. Response rate for the Indonesian residents was 93%. Details of the 2013 Indonesian national basic health research report has been published elsewhere ( The Ministry of Health, Republic of Indonesia, 2013b). For the purpose of this analysis, a subset of East Java participants 5 to 100 years old who participated in the survey was analyzed.

### Data collection and management

Data was collected through a questionnaire in the Indonesian language. The outcome of interest was utilization of dental services. It was self-reported by respondents by answering a single question “Have you received dental treatment(s) during the last twelve months?” The response options were yes or no.

Potential indicators of the dental service utilization in this study were socio-demographic characteristics, behavioral factors, and clinical condition, as informed by the existing literature ( Fonseca *et al.,* 2017; Machry *et al.,* 2013; Vieira *et al.,* 2019). Socio-demographic characteristics included age, sex, education and residential location. For inclusion in the analysis, the respondent had to be between 5 and 100 years old and must have completed the Riskesdas 2013 oral examination. Age was then categorized into <25, 25–<50, and ≥50 years old. The choice of the cut-off points for the age categorization was based in the distribution of the age. Sex was recorded as male or female. Education was measured by the highest level of school, post-school, or tertiary educational attainment and dichotomized into junior high school or less vs senior high school or higher. East Java province consists of 29 districts and nine municipalities, which differ due to the size of the area, capital, and development such as in the economy and education. Municipalities are usually ahead of the districts in terms of socio-capital development. Thus, residential location was dichotomized into districts and municipalities. The behavioral factor was tooth brushing habit (self-reported by the respondents as good vs bad tooth brushing habit). Respondents were categorized as having a good tooth brushing habit if they answered yes to the question “do you brush your teeth daily?”. Clinical condition was measured through dental status (self-reported by respondents as dentate [having one or more teeth] vs edentulous).

### Statistical analysis

Statistical analysis was performed using SAS version 9.4-callable SUDAAN version 11.0.3 (Research Triangle Institute, North Carolina, USA). Characteristics of the study participants were presented using descriptive statistics. Bivariate analyses of the association between utilization of dental treatment and each of the potential indicators were performed using chi square tests. The potential indicators include socio-demographic characteristics (age, sex, education and residential location), dental behavior (tooth brushing habit), and clinical (dental) condition. Multivariable logistic regression analysis was conducted to model together these factors influencing dental treatment utilization among a sample with information on all study variables (N=79,322); no imputation was done for missing data. The statistical significance of the associations was evaluated at P < 0.05.

### Ethical review

Ethical approval of Riskesdas 2013 was granted by the Ministry of Health Republic of Indonesia’s Human Research Ethics Committee. However, this particular study involved secondary analysis of anonymized data, and no new ethical clearance was required.

## Results

Table 1 shows the characteristics of the total study participants and the final participants included in the multivariable analysis. From a total of 90,551 respondents, 79,322 respondents with information on all variables were included in the final analysis. The included respondents were similar in all characteristics to the total number of respondents except in the age and educational level. Those included in the final analysis had a higher education level when compared with the total respondent population.

**Table 1.** Demographic and background characteristics of the study participants.

Characteristics	Total respondents		Total respondents with information on all study variables (final analysis population)	
	Size (n)	% of respondents [95% CI]	Size (n)	% of respondents [95% CI]
	N=90,551		N=79,322	
Outcome				
Dental services utilization				
Received dental treatment during the last 12 months	8,157	9 [8.8-9.2]	6,820	8.6 [8.4-8.8]
Did not receive dental treatment during the last 12 months	82,394	91 [90.8-91.2]	72,502	91.4 [91.2-91.6]
Indicators				
Socio-demographic characteristics				
Age *				
<25 years old	28,927	31.9 [31.6-32.2]	17,698	22.3 [22.0-22.6]
25–<50 years old	35,817	39.6 [39.2-39.9]	35,817	45.2 [44.8-45.5]
≥50 years old	25,807	28.5 [28.2-28.8]	25,807	32.5 [32.2-32.9]
Sex				
Male	43,570	48.1 [47.8-48.4]	37,694	47.5 [47.2-47.9]
Female	46,981	51.9 [51.6-52.2]	41,628	52.5 [52.1-52.8]
Education *				
Junior high school or less	68,925	77.4 [77.1-77.6]	59,160	74.6 [74.3-74.9]
Senior high school or higher	20,162	22.6 [22.4-22.9]	20,162	25.4 [25.1-25.7]
Residential place				
Districts	73,617	81.3 [81.0-81.6]	64,562	81.4 [81.1-81.7]
Municipality	16,934	18.7 [18.4-19.0]	14,760	18.6 [18.3-18.9]
Oral health behavior				
Tooth brushing habit				
Self-reported bad tooth brushing habit	6,111	7.4 [7.2-7.6]	6,019	7.6 [7.4-7.8]
Self-reported good tooth brushing habit	76,601	92.6 [92.4-92.8]	73,303	92.4 [92.2-92.6]
Clinical condition				
Dental Status				
Dentate	77,191	97.3 [97.2-97.4]	77,191	97.3 [97.2-97.4]
Edentulous	2,131	2.7 [2.6-2.8]	2,131	2.7 [2.6-2.8]

95% CI: 95% Confidence Interval; *Significant different

The results from the bivariate analyses are presented in Table 2. All the indicators showed a significant relationship with the utilization of dental services. In terms of age, people over 50 years old showed the lowest utilization of dental treatment, followed by people less than 25 years old. Respondents that received the highest number of dental treatments were people between 25 and 50 years old. Furthermore, males, people with lower educational background, residents of districts, people reporting bad tooth brushing habit, and those with edentulism showed lower utilization of dental treatments compared to their counterparts.

**Table 2.** Bivariate analysis of the association between dental service utilization and each of the explanatory variables.

Indicators	Utilization of dental services (received dental treatment during the last 12 months)	
	% of respondents [95% CI]	PR [95% CI]
Socio-demographic characteristics		
Age		
<25 years old	8.4 [8.1–8.8]	**0.83 [0.79–0.87]**
25–<50 years old	10.5 [10.2–10.8]	**01.40 [1.35–1.45]**
≥50 years old	7.5 [7.2–7.9]	ref (1)
Sex		
Male	8.1 [7.9–8.4]	**00.80 [0.78–0.83]**
Female	9.8 [9.5–10.1]	ref (1)
Education		
Junior high school or less	8.7 [8.5–8.9]	**00.82 [0.79–0.85]**
Senior high school or higher	9.9 [9.5–10.4]	ref (1)
Residential place		
Districts	8.7 [8.5–8.9]	**00.87 [0.84–0.91]**
Municipality	10.2 [9.7–10.6]	ref (1)
Oral health behavior		
Tooth brushing habit		
Self-reported bad tooth brushing habit	4.1 [3.6–4.6]	**0.45 [0.41–0.48]**
Self-reported good tooth brushing habit	9.0 [8.8–9.2]	ref (1)
Clinical condition		
Dental status		
Dentate	8.7 [8.5–8.9]	**2.70 [2.32–3.15]**
Edentulous	3.2 [2.5–4.0]	ref (1)

**Bold**: indicator was significant; 95% CI: 95% Confidence Interval; PR: Prevalence Ratio; Bivariate analysis was conducted using chi-square

The results of the multivariable model are presented in Table 3. The model showed that dental services utilization differed by age. Respondents less than 25 years of age received lower dental treatment than respondents ≥50 years old (PR [95% CI] = 0.74 [0.71–0.78]). However, respondents 25–<50 years old showed more utilization of dental treatments than respondents ≥50 years old (PR [95% CI] = 1.23 [1.18–1.28]). Being male and having lower education were indicators for having a lower utilization of dental treatment (PR [95% CI] = 0.81 [0.79–0.84] and PR [95% CI] = 0.89 [0.86–0.93], respectively). Similarly, people living in districts showed lower utilization of dental treatment than those living in municipalities. Respondents with bad tooth brushing habit showed lower utilization of dental treatment. Having teeth was associated higher utilization of dental treatment (PR [95% CI] = 1.39 [1.16–1.66]).

**Table 3.** Multivariable analysis of dental services utilization.

Characteristics	Utilization of dental services (received dental treatment during the last 12 months)	
	PR	[95% CI]
**N=**79,322		
Socio-demographic characteristics		
Age		
<25 years old	**0.74**	**[0.71**– **0.78]**
25–<50 years old	**1.23**	**[1.18**– **1.28]**
≥50 years old	ref (1)	-
Sex		
Male	**0.81**	**[0.79**– **0.84]**
Female (reference)	ref (1)	-
Education		
Junior high school or less	**0.89**	**[0.86**– **0.93]**
Senior high school or higher (reference)	ref (1)	-
Residential place		
Districts	**0.91**	**[0.88**– **0.95]**
Municipality (reference)	ref (1)	**-**
Oral health behavior		
Tooth brushing habit		
Self-reported bad tooth brushing habit	**0.53**	**[0.48**– **0.59]**
Self-reported good tooth brushing habit (reference)	ref (1)	-
Clinical conditions		
Dental status		
Dentate	**1.39**	**[1.16**– **1.66]**
Edentulous (reference)	ref (1)	**-**

**Bold**: indicator was significant; 95% CI: 95% Confidence Interval; PR: Prevalence Ratio; Multi variable analysis was conducted using logistic regression

## Discussion

This study showed that the utilization of dental services by East Java residents in Indonesia was very low. Only 9% of the East Java population received dental treatments during the last 12 months, which is slightly higher than the national average (8.1%) ( The Ministry of Health, Republic of Indonesia, 2013b). Dental service utilization was low among respondents who were less than 25 years old, male, district residents, edentulous, and had lower education and poor toothbrushing habit.

Among Indonesians, the fact that 95.5% of the population never utilize dental services was revealed in the 2018 national health survey ( The Ministry of Health. Republic of Indonesia, 2019). The percentage of the population that never accesses dental services in Indonesia was far higher than that has been reported in rural India, another developing country ( Gupta *et al.*, 2014). Research in a rural population of western Rajasthan, revealed that around 55% of the population never visited a dentist while only 1.5% visited a dentist in the last 6 months ( Gupta *et al.*, 2014). However, these estimates contradict some research findings from economically developed countries where approximately 40%–80% of the population have accessed dental services within the last 12 months ( Brown & Lazar, 1999; Kiyak & Reichmuth, 2005). Lack of awareness about oral health could be a reason behind the lower utilization of dental services in East Java province, Indonesia. In Indonesia, the secondary data analysis from the National Socioeconomic Survey in 2013 also showed that a high proportion of the Indonesian people had no perceived need for dental services (98.36%) and had never utilized dental services, resulting in considerable unmet need for dental treatments (97.7%). Of those who had unmet need for dental treatments, 94.8% had no perceived need for dental services ( Malik *et al.*, 2020). Perceived need for dental care is one of the best predictors of dental service utilization ( Gibson, 2013). Individuals’ perceived dental health may influence their perceived need for dental care ( Morin *et al.*, 2005). In general, people will not attend health services unless they have health problems, and they demand health care as they believe that health service can solve their health problems ( Wright *et al.*, 1998).

In East Java, utilization of dental services varied according to socio-demographic, behavioural, and clinical factors. People aged 25–<50 years had the highest utilization of dental services in this study, followed by those ≥50 year old. Those less than 25 years old had the lowest utilization of dental treatment. Among Indonesians, people in 25–<50 years of age are considered the productive workforce, at which point they have usually progressed in their careers to a point and are earning enough to allow them to have insurance or utilize dental services privately. This could be the reason for greater utilization of dental treatment in this age group. The lowest utilization of dental services among people less than 25 years old could also be influenced by dental anxiety. Previous study in the Indonesian population reveals that people less than 25 years old have higher dental anxiety than their counterparts ( Prihastari *et al.*, 2020). People aged younger than 25 years old still had limited self-control and were less focussed on long-term consequences relative to people aged 25 years and older. These characteristics lead to an increased risk of unhealthy behaviours among people in this age group ( Committee on Improving the Health, Safety, and Well-Being of Young Adults, 2015), including the lack of dental service utilization to maintain their oral health. However, high levels of self-concept among people aged 25 and older may help them maintain their oral health and motivate them to improve dental service utilization. Similarly, for baby boomers (people aged 50 years and older), physical attractiveness is important, as evidenced by the growing number of people in this age group seeking cosmetic dental care ( Kiyak, 2015).

This study finds that females have greater utilization of dental services, supporting previous studies ( Emerich *et al.*, 2015; Fonseca *et al.,* 2017; Green & Pope, 1999; Honkala *et al.*, 1997; Saintrain *et al.*, 2014), while also contradicting another study ( Akbar *et al.*, 2019). Women’s greater use of oral health service providers is likely because they pay more attention to esthetics and oral hygiene. Research shows that women pay more attention to their appearance and health ( Green & Pope, 1999). On the other hand, men tend not to seek dental service due to the lack of perception of their need ( Kiyak, 1993). However, each gender’s perspectives and beliefs may differ culturally, explaining the contradictory results. Higher dental service use was also observed among working women than working men in a study in Japan ( Nishide *et al.*, 2017). One possible explanation is that working women were more likely than working men to allocate time to use dental service. Compared to men, women had a greater interest in health and literacy and put more attention to oral health ( Kawamura *et al.*, 1999).

Education is also a significant contributing factor to dental service utilization. People with lower educational background have lower access to dental treatment, supporting previous research. Considering that the analysis sample in this study were better educated than the total respondent population only underestimates this problem. A study showed that higher educated individuals visited the dentist 10 times more than those with low education ( Barros & Bertoldi, 2002). A previous study has demonstrated a higher level of oral health knowledge among people with a high educational background than those with low educational background. Having adequate oral health knowledge is attributed to positive attitudes towards dental service utilization, resulting in more educated people using dental services than less educated people ( Zhu *et al.*, 2005). Our results indicate that prevention programs would benefit by specifically targeting less educated people.

East Java province is one of the provinces located in the main island, Java. Among other islands, the Java island is categorized as the most developed. Thus, socioeconomic inequality among each area in East Java could be considered lower than other less developed islands. However, this research still finds that dental service utilization is lower among the district residents than the municipality residents in East Java. This finding supports previous research ( Akbar *et al.*, 2019). It is known that public transport networks are less developed in rural areas than in urban areas. Lack of transport access to dental facilities can be an obstacle to routine dental visit ( Hamano *et al.*, 2017; Ogunbodede *et al.*, 2015). Moreover, the use of a service also depends on the perception of user’s needs, influenced by their values, beliefs and cultures ( Giordani *et al.*, 2010). A study in Istanbul ( Ozkan *et al.*, 2011) found that more than half of the population surveyed did not feel the need or have the desire to visit a dentist, although their dental conditions were not ideal.

This study finds that people who have bad tooth brushing habit utilize dental services less, supporting previous research ( Jordao *et al.*, 2018). Jessor’s problem behaviour theory ( Jessor, 1991) proposes that various risk behaviours are inter-related. Research ( Jordao *et al.*, 2018) has affirmed this theory in the dental behaviors field, finding that there are clustering patterns in dental health behaviors whereby less frequent tooth brushing is clustered with high sugar intake, current smoking, and lack of dental visits.

Moreover, dentate people in East Java utilize more dental services than edentulous people, similar with other research finding ( Tuominen, 1987). In many countries, the reason for dental service use is to mainly undergo dental treatment. This is especially true in developing countries where most of the people visit dental care services only when they are in pain ( Varenne *et al.*, 2006). Edentulousness in some parts of the world has been thought of as a healthy condition without pain even though this condition could reduce the ability to chew certain types of food, reducing the quality of life. Elders were also found to be more resilient to poor clinical status compared to younger people ( Slade & Sanders, 2011). The importance of dentate status in predicting dental service utilization is evident. It has also been argued that age will not be a significant predictor of dental service utilization if individuals were grouped by dentate status ( Gibson, 2013).

The strength of this study lies in the nature of data collected in a national survey, allowing a representative data of the East Java population. The cross-sectional study design is a limitation as it precludes causal explanations. Self-reported utilization of dental services and lack of data on insurance coverage and income could be other limitations of this study as associations between lower socioeconomic status and decreased access to dental services have been found in several countries ( Grytten *et al.*, 2012; Hjern *et al.*, 2001; Larson & Halfon, 2010; Listl, 2011; Murakami *et al.*, 2014; Tchicaya & Lorentz, 2014). Some potential bias could arise due to self-reported data and residual confounder. However, the study findings are in agreement with prior research that assessed dental care utilization, and it provides avenues for future research. This study has the potential to inform guidelines, or specific changes to existing policy or practice in Indonesia. Recognizing significant indicators that influence dental care utilization may aid in the planning and provision of programs for dental services and resource allocation. Strategies for the improvements in dental care utilization may require a multidimensional approach. The results may also inform oral health practitioners and policymakers about specific target groups requiring oral health intervention programs.

## Conclusions

This first detailed population-based study in the East Java province of Indonesia has demonstrated that the use of dental services is influenced by socio-demographic factors. People less than 25 years old, male, those with lower educational background, those living in a district, those with poor tooth brushing habit and being edentulous are associated with lower dental service utilization.

## Data availability

Data used for this analysis are available by a written request to the Ministry of Health Republic of Indonesia.

### Source data

The available variables of Riskesdas 2013 dataset could be learnt from the national report of Riskesdas 2013 produced by Indonesian Ministry of Health, available online in http://labdata.litbang.kemkes.go.id/images/download/laporan/RKD/2013/Laporan_riskesdas_2013_final.pdf. A written request of a sub data set should be sent to the Ministry of Health Republic of Indonesia (sent to the head of research and development division of the Ministry of Health Republic of Indonesia at Jl. Percetakan Negara no 29 Jakarta Pusat, Indonesia) along with a proposal detailing the proposed analysis. After approval, the proposal will be analyzed by the data management laboratory. Successful applicants will get the data by email after signing a letter of agreement about the data management, including an agreement to neither send the data to other party nor using it for other reason than that has been agreed by the Ministry. The instruction of how to apply for the data are available from the Indonesian Ministry of Health’s website: http://labmandat.litbang.kemkes.go.id/images/download/peraturan/alur.pdf
