# Supplementary material for: Factors influencing the utilization of dental services in East Java, Indonesia
Source: F1000Res. 2021 Apr 21;9:673. Originally published 2020 Jul 3. [Version 2] doi: 10.12688/f1000research.23698.2 (PMC8082568; doi:10.12688/f1000research.23698.2)
Supplement: Peer review history

# Peer review: Reviewer response for version 2

**DOI:** 10.5256/f1000research.55792.r83675

**Published:** 2021-04-28

**Authors:** A. Gupta

**Affiliations:** 1 Deakin University, Geelong, Vic, Australia

No further comments. Thank you.

Is the work clearly and accurately presented and does it cite the current literature?

Partly

If applicable, is the statistical analysis and its interpretation appropriate?

Yes

Are all the source data underlying the results available to ensure full reproducibility?

Yes

Is the study design appropriate and is the work technically sound?

Yes

Are the conclusions drawn adequately supported by the results?

Yes

Are sufficient details of methods and analysis provided to allow replication by others?

Yes

Reviewer Expertise:

Public health, oral health

I confirm that I have read this submission and believe that I have an appropriate level of expertise to confirm that it is of an acceptable scientific standard.

# Peer review: Reviewer response for version 1

**DOI:** 10.5256/f1000research.26147.r70071

**Published:** 2020-09-11

**Authors:** A. Gupta

**Affiliations:** 1 Deakin University, Geelong, Vic, Australia

This is a very interesting paper but has some inconsistencies throughout. First, it is unclear whether the paper focusses on exploring factors influencing HIGH or LOW (or both) utilization of dental services in East Java, Indonesia. The background makes a case for LOW utilisation but the title and the overall conclusion state otherwise. Further, the discussion section is very superficial and will benefit from a thorough discussion of the study findings with the existing evidence. Some specific comments for the authors to consider are as follows:   

 Specific comments –

**Title: **Clarify whether the paper focuses on exploring factors influencing HIGH or LOW (or both) utilisation of dental services in East Java, Indonesia

**Background:**

- 3 ^rd^ paragraph: Support the sentences with more and relevant evidence.
- 5 ^th^ paragraph:
  - In the sentence, ‘Despite…..2013b)’ do you mean ‘use of dental treatment’ or ‘UTILIZATION OF DENTAL SERVICES’? Some clarity will be helpful.
  - In the sentence “However…..2013b)”, the phrase ‘treat utilization for dental issues’ needs clarity.
  - Some discussion on the QUALITY of the public dental service will be helpful. Further, some statistics on ‘PRIVATE DENTAL CARE UTILIZATION’ as compared to the public dental service will be helpful.
  - Revise the study aim for clarity- whether the paper aims to explore factors influencing HIGH or LOW (or both) utilization of dental services in East Java, Indonesia.

**Methods:**

- Page 4, 2 ^nd^ line: “Indicators of…..condition” seems inappropriate as it is meant to be the findings of this study. Suggest either deleting this sentence or adding the word ‘POTENTIAL’ at the start of this sentence. It will also be good if the authors can support this sentence with evidence, assuming that their choice of exposure variables was informed by previous literature.
- In the same paragraph as above- in the sentence ‘East Java province….. districts), it will be helpful if the authors can expand on what they mean by ‘where municipalities are usually ***ahead*** of the districts’.
- Statistical Analysis: Authors mention ‘no imputation was done for missing data’ but no information (eg., n =?)  on the missing data is provided. Please include the necessary information. Also, justification on how was missing data treated if no imputation was done will be helpful.

**Results:**

- Please insert the total sample size (n) in Table 1.

**Discussion:**

- Overall the discussion section in its current form is weak and superficial. It might be worth including a thorough and integrated discussion of the findings with the existing literature.
- Page 5: Sentences such as “Lack of awareness about oral health could be a reason behind the lower utilization of dental services” will be worth discussing a bit more.
- Can authors include a bit more discussion in each of the paragraphs below:
  - In the paragraph that talks about the difference in AGE for the utilisation- authors can include some discussion on Dental anxiety as one of the potential reasons for low utilisation among participants <25 years.
  - In the paragraph that talks about the GENDER difference for the utilisation- some more discussion on why there exists contradictory evidence will be helpful. Also supporting evidence stated is primarily from developed countries. Is there any evidence from developing countries that can support the justification- given that women's perspectives and beliefs may differ culturally?
- Suggest including similar discussions in other paragraphs on education, health behaviours, etc.
- In the limitations section, it will be helpful if the authors can include some discussion on the potential of bias arising due to self-reported data and residual confounders.
- A paragraph on the research and policy implications of this study is missing and will be worth including. This study has the potential to inform guidelines, or specific changes to existing policy or practice in Indonesia.

**Conclusion:**

- A clear and precise conclusion will be helpful.
- Please clearly state whether age (what age group?), sex (male/female), education (low/high), and residential location (district/municipal) are indicators of low utilization.

Is the work clearly and accurately presented and does it cite the current literature?

Partly

If applicable, is the statistical analysis and its interpretation appropriate?

Yes

Are all the source data underlying the results available to ensure full reproducibility?

Yes

Is the study design appropriate and is the work technically sound?

Yes

Are the conclusions drawn adequately supported by the results?

Yes

Are sufficient details of methods and analysis provided to allow replication by others?

Yes

Reviewer Expertise:

Public health, oral health

I confirm that I have read this submission and believe that I have an appropriate level of expertise to confirm that it is of an acceptable scientific standard, however I have significant reservations, as outlined above.

# Response

**Published:** 2021-04-03

**Authors:** Ninuk Hariyani

**Affiliations:** Airlangga University, Indonesia

**Reviewer: 2.** **A. Gupta**

 University of Melbourne, Melbourne, Vic, Australia

**Comment 1:-** This is a very interesting paper but has some inconsistencies throughout.

*Response:-* Thank you for your constructive feedback on our manuscript. Point by point responses to your comments is provided.

**Comment 2:-** **Title: **Clarify whether the paper focuses on exploring factors influencing HIGH or LOW (or both) utilisation of dental services in East Java, Indonesia

*Response:-* The objective of this study was to see which of the included sociodemographic, behavioural, and clinical factors influenced the existing dental service utilisation. The study does not differentiate between low and high utilisation as the overall utilisation is low. To clarify this, we have detailed the study objectives in the abstract and the introduction.

**Comment 3:-** **Background: **3rd paragraph: Support the sentences with more and relevant evidence.

*Response:-* Some references have been added to support and extend the topic.

**Comment 4:-** **Background: **5th paragraph: In the sentence, ‘Despite…..2013b)’ do you mean ‘use of dental treatment’ or ‘UTILIZATION OF DENTAL SERVICES’? Some clarity will be helpful.

*Response:-* The sentence has been revised.

**Comment 5:-** **Background: **In the sentence “However…..2013b)”, the phrase ‘treat utilization for dental issues’ needs clarity.

*Response:-* Thank you for your comments. The consistent term of the utilization of dental services was used to reduce the ambiguity.

**Comment 6:-** **Background: **Some discussion on the QUALITY of the public dental service will be helpful. Further, some statistics on ‘PRIVATE DENTAL CARE UTILIZATION’ as compared to the public dental service will be helpful.

*Response:-* Some discussion has been added.

**Comment 7:-** **Background: **Revise the study aim for clarity- whether the paper aims to explore factors influencing HIGH or LOW (or both) utilization of dental services in East Java, Indonesia.

*Response:-* The aim of this study was to see which of the included sociodemographic, behavioural, and clinical factors influenced the existing dental service utilisation. The study does not differentiate between low and high utilisation as the overall utilisation is low. To clarify this, we has detailed the study objectives in the abstract and the introduction.

**Comment 8:-** **Methods: **Page 4, 2nd line: “Indicators of…..condition” seems inappropriate as it is meant to be the findings of this study. Suggest either deleting this sentence or adding the word ‘POTENTIAL’ at the start of this sentence. It will also be good if the authors can support this sentence with evidence, assuming that their choice of exposure variables was informed by previous literature.

*Response:-* Thank you for the suggestion. It has been adopted and the changes have been made.

**Comment 9:-** **Methods: **In the same paragraph as above- in the sentence ‘East Java province….. districts), it will be helpful if the authors can expand on what they mean by ‘where municipalities are usually ***ahead ***of the districts’.

*Response:-* Explanation has been added.

**Comment 10:-** **Methods: **Statistical Analysis: Authors mention ‘no imputation was done for missing data’ but no information (eg., n =?) on the missing data is provided. Please include the necessary information. Also, justification on how was missing data treated if no imputation was done will be helpful.

*Response:-* The total sample size of the final analysis has been added both in the method and Table 3 /multivariable analysis. The comparison of the characteristics of the total study participants and the final participants included in the multivariable analysis was also added in table 1 and the first paragraph of the results section. The included respondents were similar in all characteristics to the total number of respondents except in the educational level. The implication of this reduced sample size to the general finding has also has been added in the discussion. While previous article showed that people with lower educational background have lower access to dental treatment, this new article showed that due to the fact that the analysis sample in this study were better educated than the total respondent population, the influence of education on dental service utilization might only be underestimated.

**Comment 11:-** **Results: **Please insert the total sample size (n) in Table 1.

*Response:-* The sample size has been added as an additional column. We also provided a comparison of the characteristics of the total study participants and the final participants included in the multivariable analysis

**Comment 12:-** **Discussion: **Overall the discussion section in its current form is weak and superficial. It might be worth ****including a thorough and integrated discussion of the findings with the existing literature.

*Response:-* The discussion has been expanded in each of the suggested sections.

**Comment 13:-** **Discussion: **Page 5: Sentences such as “Lack of awareness about oral health could be a reason behind the lower utilization of dental services” will be worth discussing a bit more.

*Response:-* The discussion has been expanded.

**Comment 14:-** **Discussion: **Can authors include a bit more discussion in each of the paragraphs below: In the paragraph that talks about the difference in AGE for the utilisation- authors can include some discussion on Dental anxiety as one of the potential reasons for low utilisation among participants <25 years.

*Response:-* Discussion and literature on dental anxiety has been added.

 The discussion for each paragraph has been expanded.

**Comment 15:-** **Discussion: **Can authors include a bit more discussion in each of the paragraphs below: In the paragraph that talks about the GENDER difference for the utilisation- some more discussion on why there exists contradictory evidence will be helpful. Also supporting evidence stated is primarily from developed countries. Is there any evidence from developing countries that can support the justification- given that women's perspectives and beliefs may differ culturally?

*Response:-* The discussion has been expanded.

**Comment 16:-** **Discussion: **Suggest including similar discussions in other paragraphs on education, health behaviours, etc.

*Response:-* The discussion has been expanded.

**Comment 17:-** **Discussion: **In the limitations section, it will be helpful if the authors can include some discussion on the potential of bias arising due to self-reported data and residual confounders.

*Response:- *The discussion has been expanded.

**Comment 18:-** **Discussion: **A paragraph on the research and policy implications of this study is missing and will be worth including. This study has the potential to inform guidelines, or specific changes to existing policy or practice in Indonesia.

*Response:-* The discussion has been expanded.

**Comment 19:-** **Conclusion: **A clear and precise conclusion will be helpful.

*Response:- *The discussion has been expanded.

**Comment 20:-** **Conclusion: **Please clearly state whether age (what age group?), sex (male/female), education (low/high), and residential location (district/municipal) are indicators of low utilization.

*Response:- *The discussion has been expanded.

# Peer review: Reviewer response for version 1

**DOI:** 10.5256/f1000research.26147.r66387

**Published:** 2020-07-16

**Authors:** Azlan Jaafar

**Affiliations:** 1 Department of Periodontology & Community Oral Health, Faculty of Dentistry, Universiti Sains Islam, Nilai, Malaysia

The article is scientifically-well-written. No major issues related to methods or data analysis need to be highlighted. The authors have indicated the problem statement in the introduction on why they conduct the study. However, comments were given to improve the flow and content of the section.

 The method is well-describe. The analysis performed is matched with the study design as recommended by others. The outcome measure of dental service utilization is following a well-versed definition by other researchers. However, there are some categorisations of independent variables that do not match the results. For example the age and dental status. The author stated that they are using ≤25, 25-<50, and ≥50 years old. However, in the results it was mentioned differently (less than 25, 25-50, and ≥50. The age categorisation should not overlap thus I would like to suggest new categorisation <25, 25-50, and >50. Whereas, the dental status was informed in methods was “dentate vs edentulous” but in the results it mentioned, “not edentulous vs edentulous”. Although it seems to give similar meaning but consistency in reporting is essential.

 The rest including abstract, discussion, and conclusion is well-written. The citation provided is also updated evidence. I also have given some papers for citations to enrich the discussion part. Other related information such as data sources is also stated in the identification of originality of the data.  

Please see this annotated pdf.

Is the work clearly and accurately presented and does it cite the current literature?

Yes

If applicable, is the statistical analysis and its interpretation appropriate?

Yes

Are all the source data underlying the results available to ensure full reproducibility?

Yes

Is the study design appropriate and is the work technically sound?

Yes

Are the conclusions drawn adequately supported by the results?

Yes

Are sufficient details of methods and analysis provided to allow replication by others?

Yes

Reviewer Expertise:

Dental Public Health

I confirm that I have read this submission and believe that I have an appropriate level of expertise to confirm that it is of an acceptable scientific standard.

# Response

**Published:** 2021-04-03

**Authors:** Ninuk Hariyani

**Affiliations:** Airlangga University, Indonesia

**Reviewer(s)' Comments to Author:**

**Reviewer: 1.** **Azlan Jaafar**

 Department of Periodontology & Community Oral Health, Faculty of Dentistry, Universiti Sains

 Islam, Nilai, Malaysia

**Comment 1:-** The article is scientifically-well-written. No major issues related to methods or data analysis need to be highlighted. The authors have indicated the problem statement in the introduction on why they conduct the study.

*Response:-* Thank you for your constructive feedback on our manuscript.

**Comment 2:-** The method is well-describe. The analysis performed is matched with the study design as recommended by others. The outcome measure of dental service utilization is following a well-versed definition by other researchers.

*Response:-* Thank you for your constructive feedback on our manuscript.

**Comment 3:-** There are some categorisations of independent variables that do not match the results. The author stated that they are using ≤25, 25-<50, and ≥50 years old. However, in the results it was mentioned differently (less than 25, 25-50, and ≥50. The age categorisation should not overlap thus I would like to suggest new categorisation <25, 25-50, and >50.

*Response:-* Thank you for the correction. The categorisations of < 25, 25-<50, and ≥50 has been used throughout the text except the one you pointed out. The change has been made to make it consistent throughout the manuscript.

**Comment 4:-** The dental status informed in methods was “dentate vs edentulous” but in the results it mentioned, “not edentulous vs edentulous”. Although it seems to give similar meaning but consistency in reporting is essential.

*Response:-* Thank you for the suggestion. The change of “dentate vs edentulous” has been made in Table 1, Table 2 and Table 3.

**Comment 5:-** The rest including abstract, discussion, and conclusion is well-written. The citation provided is also updated evidence.

*Response:-* Thank you for your constructive feedback on our manuscript.
